# Mental health interventions for humanitarian volunteers: a scoping review

**DOI:** 10.1136/bmjopen-2024-095363

**Published:** 2025-07-06

**Authors:** Sarker Mohammad Nasrullah, Tanjamul Refat, Martina Emelia Gustavsson

**Affiliations:** 1 Department of Global Public Health, Karolinska Institutet, Stockholm, Sweden; 2 Department of Public Health, North South University, Dhaka, Bangladesh; 3 Department of Public Health, Northern University Bangladesh, Dhaka, Bangladesh

**Keywords:** MENTAL HEALTH, Psychosocial Intervention, Review

## Abstract

**Abstract:**

**Objectives:**

The aim of this scoping review was to map the nature and extent of the existing literature on mental health interventions for humanitarian volunteers in disaster contexts. The study also explored how the interventions were evaluated.

**Design:**

The methodology of this scoping review followed the extended guidelines of the Preferred Reporting Items for Systematic Reviews and Meta-Analyses for Scoping Reviews.

**Data sources:**

Five academic bibliographic databases (PubMed, Embase, Web of Science, EBSCOhost and Google Scholar), grey literature websites (Google Scholar, ProQuest, Policy Commons, etc.) and relevant organisational archives were systematically searched for eligible documents.

**Eligibility criteria:**

Both peer-reviewed and grey literature studies on mental health interventions for humanitarian volunteers in the context of any type of disaster were eligible for inclusion. Research papers that evaluated any such intervention were also included. Documents that targeted professional humanitarian workers or explored physical health conditions or diseases in disaster contexts, letters to the editor, comments, correspondence and research protocols were excluded. There were no restrictions in terms of the date and language of the documents.

**Data extraction and synthesis:**

A systematic search of the targeted databases was conducted from 12 May 2025 to 20 May 2025. Deduplication, screening and full-text evaluation for the selection of documents were done using the online version of Rayyan. Data were collected and recorded into a structured Microsoft Excel sheet. Two researchers individually conducted the selection of the articles and the extraction of data. A third researcher helped to resolve any discrepancies if required.

**Results:**

A total of 2627 documents were retrieved by searching the targeted databases and websites. After matching them with the eligibility criteria, 20 documents were included in the final list. 14 of them were research papers; the rest was organisational literature. All the papers were from 2006 and later, except one that was from 1998. No documents were found from the Middle East, North Africa and Sub-Saharan regions. 10 broad categories of interventions were identified, which were either implemented in the field or suggested in the form of guidelines. Most of the interventions were postexposure and preventive in nature. Psychological first aid was the most widely used intervention in this context, being used by the national societies of the International Federation of Red Cross and Red Crescent Societies. Nine of the documents were research papers evaluating the effectiveness of the interventions using different scales and customised questionnaires. Four of them did not observe any notable effect on the mental health of the participants.

**Conclusions:**

Over the past two decades, the evidence on mental health interventions for humanitarian volunteers has grown. The reviewed literature documented various interventions and guidelines that need further study and testing to both prove and improve their effectiveness. Organisational policies could incorporate and further evaluate these to ensure the psychosocial well-being of volunteers. A review of research papers on intervention effectiveness found heterogeneity in settings, designs, interventions and methods, precluding a systematic review. More research is needed on individual interventions, volunteer perceptions and comparing interventions to identify the most effective ones. Additionally, comparing pre-exposure and postexposure interventions with multimodal systems that support volunteers throughout deployment is recommended.

**STRENGTHS AND LIMITATIONS OF THIS STUDY**

- The search for literature studies was not restricted by time, geography or language, thereby enhancing its strength to achieve the aim of this scoping review.
- The review had a broad scope, including all types of interventions in any type of disaster context, which helped to capture a comprehensive overview of the topic.
- The small number of documents included and missing data from important geopolitical regions raise questions about the findings’ generalisability.
- This research did not solicit expert opinions or insights into the topic under discussion.
- The review did not investigate the effectiveness of the interventions in question.

## Background

 According to the Global Natural Disaster Assessment Report by the United Nations Office for Disaster Risk Reduction (UNDRR), 326 major nature-related disasters affected 117 countries worldwide in 2023.^1^ A 2023 report by the Food and Agriculture Organisation estimated the number to be around 400 per year in the past 20 years.^2^ The numbers are expected to only rise in the future due to climate change effects.^2 3^ In addition to that, the world has seen at least 80 armed conflicts of different categories and intensities per year since 2001, adding to the distress, devastation and death among people.^4^ Disasters, whether nature-related or human-induced, overwhelm the local capacities in most cases. Most often, the affected communities are forced to seek external assistance in the form of human, financial or material resources.^5^ Community-based organisations and civil societies primarily distribute these aids through their professional staff and volunteers, using their knowledge and skills as local actors in effective disaster response.^6^

Volunteers are valuable community stakeholders in all kinds of settings. An estimate shows that approximately 970 million volunteers in total are working globally in different sectors, including humanitarian sectors, around 25% of whom contribute their time and skills through various types of organisations.^7^ Among the international non-governmental organisations, the United Nations and the International Federation of Red Cross and Red Crescent Societies (IFRC) are the two major organisations that depend on volunteers in the fields. The United Nations Volunteers (UNV) deployed 12408 volunteers in 2022 alone to contribute in the areas of development, peace, security and human rights.^8^ These volunteers also work in protracted crises, such as the postearthquake condition in Haiti, the Rohingya refugee crisis in Bangladesh and similar situations in 40 more countries.^8^ On the other hand, the IFRC is the world’s largest humanitarian network, and it has been gradually developing its national offices around the globe for the last 150 years. There are currently Red Cross and Red Crescent national societies in 191 different countries, supervising a total of 16 million volunteers locally.^9 10^ In addition to disaster response, these volunteers remain active before and after disaster events, raising awareness and building community capacities and resilience.^10^ The smaller, local and national volunteer organisations and groups also play equally essential roles in their respective contexts.

Both nature-related and human-induced disasters contain potential risk factors for adverse mental health consequences such as major depressive disorder, post-traumatic stress disorder (PTSD), substance use disorders, and somatoform disorders.^11^ According to the WHO, almost the entire population affected by a disaster faces the risk of experiencing psychological distress at some point in time.^12^ And in the case of armed conflicts, approximately 22% of people living in affected communities may develop depression, anxiety, PTSD, bipolar disorder or schizophrenia within the next 10 years.^13^ A systematic review and meta-analysis on data from 22 studies involving 48170 participants linked ill mental health outcomes in the affected populations after disasters.^14^ For example, one of these studies was conducted on flood-affected people in Hunan, China, diagnosing 2336 (9.2%) participants as probable PTSD-positive.^15^

Likewise, humanitarian aid workers responding to disaster-affected communities have shown signs of acute stress, burnout, compassion fatigue, harmful alcohol consumption and suicidal ideation.^16^,^19^ In fact, a study shows that they could be at greater risk of adverse psychological effects compared with the general population.^20^ In the course of their work, they face potentially traumatic events (PTEs), long working hours, harsh working conditions and interpersonal conflicts while living away from their families.^20^,^23^ Furthermore, in the context of armed conflicts, humanitarian staff are prone to similar safety and security concerns as the affected population. In 2022, 143 aid workers sustained injuries, 185 faced kidnapping and 116 lost their lives during deployment.^24^ According to a review of studies on humanitarian workers’ mental health, the prevalence of PTSD ranged from approximately 6% to 42% in different countries and contexts. Most of the studies included in this review were from Asia and Africa, encompassing national humanitarian staff, volunteers and expatriates.^22^ Another systematic review observed the burden of psychological distress (6.5%–52.8%), burnout (8.5%–32%), anxiety (3.8%–38.5%), depression (10.4%–39%) and PTSD (0%–25%) among humanitarian aid workers consisting of both civilians and professionals.^25^

The effects of PTEs on humanitarian staff vary greatly depending on factors such as individual characteristics and resilience, degree of exposure, severity of the events, and social support.^17 26^ Compared with professionals, volunteers often tend to be more susceptible due to their limited access to resources, inadequate training and lack of post-deployment support.^17 26 27^ Despite their familiarity with the area and the community, the same factors could adversely affect the mental health of community volunteers, who typically come from the affected population. Moreover, they could be dealing with personal loss due to the disaster, adding to their vulnerabilities. Significant differences in mental health outcomes between community volunteers and regular staff or volunteers have been observed in two studies.^28 29^

In this context, most of the research on potential mental health interventions targeted professional first responders, including regular humanitarian staff, police, firefighters, emergency medical services, and the military. Very few studies have focused solely on volunteers, making it important to explore the available evidence. The aim of this review is to map the nature and extent of the existing literature on mental health interventions for humanitarian volunteers in disaster contexts and to explore how they were evaluated. In this regard, the flexible methodology of scoping review is suitable to conduct a comprehensive search across a heterogeneous body of literature and diverse information sources, such as electronic databases and organisational websites, including both peer-reviewed and grey literature, to have a better understanding of the matter. Moreover, the availability of data and the quality of existing evidence will help to determine the practicality of a systematic review of the topic. Thus, it will help identify the gaps in knowledge and indicate the areas where further research is needed.

## Methods

### Protocol

This scoping review does not have a published protocol. It was conducted and reported in accordance with the extended guidelines of the Preferred Reporting Items for Systematic Reviews and Meta-Analyses for Scoping Reviews (PRISMA-ScR).^30^ The PRISMA-ScR checklist has been provided as online supplemental material 1.

### Eligibility criteria

Primarily, this review aimed to search for and include documents on PTSD interventions only. However, the initial search of selected databases retrieved several potential documents that discussed interventions for other major mental health concerns in volunteers, such as stress, resilience, and psychosocial well-being. On reviewing these documents, the need for expanding the scope of this review to include them as well was recognised. Hence, the methodology of this study was revised, and a second search was conducted using an altered search strategy that targeted all types of mental health interventions. The modified eligibility criteria for the selection of relevant documents were as follows.

#### Inclusion criteria

- Documents on mental health interventions for humanitarian volunteers in the context of both nature-related and human-induced disasters were eligible for inclusion.
- Research papers that evaluated the effectiveness of such interventions and documents that suggested potential interventions in the form of guidelines were also included, even if the interventions were not implemented in the field.
- Secondary research papers were added only if they reviewed a specific intervention. Otherwise, their references were checked for relevant primary studies to be included separately.
- To observe how far back the documents date, no restrictions were set in terms of the timeframe. The selected databases were searched from inception to 20 May 2025 to include documents.
- As researchers fluent in different languages, such as French, Spanish, Arabic, and German, were available for assistance, eligible documents in any language were included.

#### Exclusion criteria

- Documents were excluded if they solely focused on professionals acting as temporary volunteers in disaster response, for example, police officers, firefighters, soldiers, medical personnel, emergency medical services, and regular humanitarian staff.
- Publications such as letters to the editor, comments, correspondence, research protocols and documents that explored physical health conditions or diseases in disaster contexts were also removed from the final inclusion list.

### Information sources

For peer-reviewed literature, the following electronic bibliographic databases were searched: PubMed (National Centre for Biotechnology Information; National Institutes of Health; Bethesda, Maryland, USA), Embase (Elsevier B.V.; Amsterdam, Netherlands) and EBSCOhost (containing CINAHL and PsycINFO records; EBSCO Information Services; Ipswich, Massachusetts, USA). Web of Science (Thomson Reuters; New York, New York, USA) and Google Scholar (Google; Mountain View, California, USA) served as sources for both peer-reviewed and grey literature.

The websites of relevant humanitarian response and disaster-related organisations were also searched for grey literature, such as: United Nations High Commissioner for Refugees (Geneva, Switzerland), ReliefWeb by the United Nations Office for the Coordination of Humanitarian Affairs (New York, New York, USA), PreventionWeb by the UNDRR (Geneva, Switzerland), International Organization for Migration (Geneva, Switzerland), IRIS (WHO; Geneva, Switzerland), International Committee of the Red Cross (ICRC; Geneva, Switzerland), IFRC (Geneva, Switzerland), Médecins Sans Frontières (MSF; Geneva, Switzerland), Emergency Events Database by the Centre for Research on the Epidemiology of Disasters (CRED; Université Catholique de Louvain; Brussels, Belgium), ProQuest (Ann Arbor, Michigan, USA), and Policy Commons (Coherent Digital; Alexandria, Virginia, USA).

### Search strategy

As mentioned earlier, the aim of this scoping review was expanded, and the methodology was revised accordingly to include all types of mental health interventions. An updated search strategy was designed from scratch, replacing the terms related to PTSD with mental health-related terms. Both old and new search results were screened to make a comparison between them and reduce the risk of missing important, relevant documents. The updated search for eligible documents was conducted between 12 May 2025 and 20 May 2025. Different keywords and their alternatives were used to search online academic databases and organisational websites. For bibliographic databases, indexed terms were combined with the keywords to build search queries using Boolean operators (AND, OR), truncation symbols and quotation symbols. The search strings had to be tailored to accommodate the slightly varying search engines of different databases. Filters were used to narrow down the search results where necessary. Both old and new search queries for different academic databases have been provided as online supplemental material 2.

### Selection of documents

The search results were imported into the web-based software Rayyan (Qatar Computing Research Institute; Doha, Qatar) to match the eligibility criteria. First, duplicates were manually removed with the assistance of the software. The documents were then subjected to a title-abstract screening, followed by a full-text evaluation for final inclusion in the review. The reference lists of all included research papers were also checked for relevant literature. Non-academic and organisational websites were screened manually to identify eligible documents. To maintain the methodological integrity of the review, all the stages of selecting documents were completed by two individual researchers. The results were then cross-matched, and the discrepancies were discussed and resolved between them. A senior researcher helped to settle any disagreements that could not be solved through discussion.

### Data extraction

A structured Microsoft Excel sheet (Microsoft Corporation; Seattle, Washington, USA) was used to chart the data. Three categories of variables were extracted from the selected literature: study characteristics, participant demographics and intervention details. General characteristics of the articles included publication type, title, author, year, location, study design, target population and sample size. Participant demographics included their number, age, affiliation, previous support and training, types of work and exposure to PTEs. Lastly, information on different interventions comprised the types of intervention, outcomes of interest, use in the field, time and mode of delivery, frequency and duration of intervention, evaluation tools and methods, findings, conclusions and recommendations. Most of the information in the organisational grey literature was limited to the specifics of the interventions. The data were extracted and cross-checked by two individual researchers. A third researcher resolved the differences between them.

### Critical appraisal and evidence synthesis

As this review aimed to map all existing literature on the topic of interest, regardless of the strength of the evidence, a critical appraisal of the included documents was not done. A narrative method of evidence synthesis was adopted to analyse and summarise the data collected. The results were presented in flowcharts, pie charts and descriptive tables in this scoping review.

### Patient and public involvement statement

Patients and/or the public were not involved in the design, conduct, reporting or dissemination plans of this research.

## Results

### Search results

Searches in different scientific bibliographic databases yielded 2627 results. After deduplication, 1413 remained for title-abstract screening. The screening process excluded 1234 documents, of which 10 could not be retrieved either because full-text articles were not available or due to faulty website links, leaving 169 for full-text evaluation. The final review included 14 peer-reviewed papers that met the eligibility criteria.

**Figure 1:**
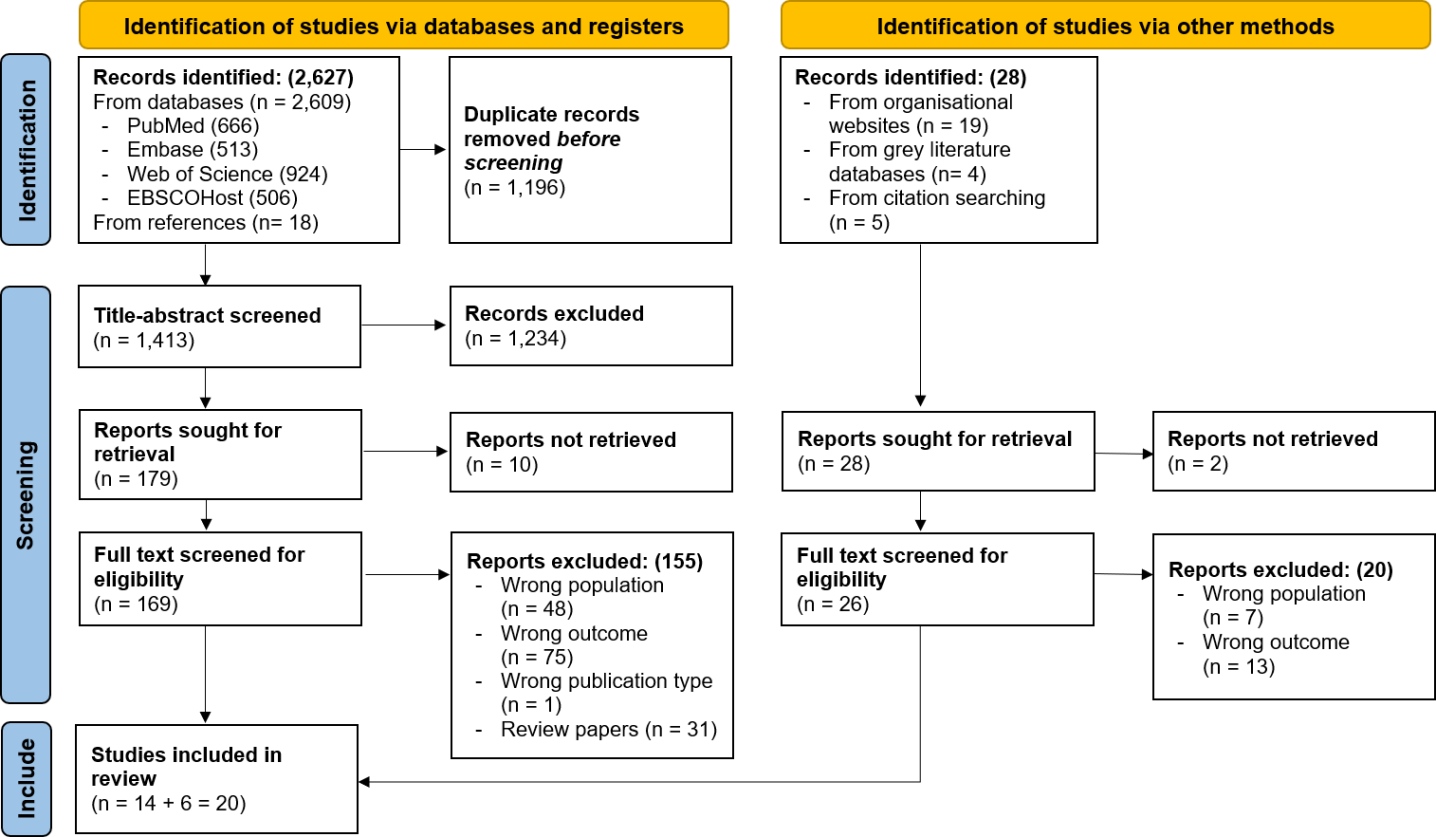
PRISMA 2020 flow diagram for this scoping review. PRISMA; Preferred Reporting Items for Systematic Reviews and Meta-Analyses.

A total of 28 records were identified from the grey literature search, of which 26 could be retrieved for a thorough evaluation. After the full text evaluation, six documents were added to the final inclusion list. Figure 1 displays the PRISMA 2020 flow diagram for the searches.

### Characteristics of the documents

The peer-reviewed papers consist of the following types of literature: three cross-sectional studies, four randomised controlled trials, one cohort study, one qualitative study, one rapid realist review, one mixed-methods study, two reports and one concept note. The grey literature included three documents on intervention guidelines, one toolkit, one workshop material and one thesis paper. Most of the documents originated between 2006 and 2024, except one from 1998.^31^ Five of them were from the USA, and one was from each of the following countries: Germany, New Zealand, Japan, Chile, Turkey, Italy, France, Australia, Indonesia and the UK. Among the documents included, 12 focused solely on volunteers, and the rest involved both volunteers and others working in the context of the following disasters: earthquake, tsunami, flood, typhoon, hurricane, volcanic eruption, displacement, terrorism and fire incidents. Table 1 presents the basic characteristics of the documents included.

**Table 1:** Basic characteristics of the included documents

Title	Author/ organisation	Year	Type	Location	Target population	Disaster context
Mindfulness meditation improves mental health in flood survivors and disaster volunteers: A randomized wait-list controlled trial*	Müller *et al*^65^	2024	Randomised controlled trial	Germany	Disaster victims and volunteers	Flood
The effect of psychological first aid intervention on stress and psychological resilience in volunteers participating in 2023 earthquakes centered in Kahramanmaraş, Turkey*	Bekircan *et al*^34^	2023	Randomised controlled trial	Türkiye	Volunteers	Earthquake
The disaster worker resiliency training program: A randomized clinical trial. *	Mahaffey *et al*^66^	2021	Randomised controlled trial	USA	Disaster workers, including volunteers	Hurricane
Group critical incident stress debriefing with emergency services personnel: A randomized controlled trial*	Tuckey and Scott^32^	2013	Randomised controlled trial	Australia	Volunteer firefighters	Fire incidents
Training to improve resilience and coping to monitor PTSD in rescue workers*	Scuri *et al*^56^	2019	Cross-sectional study	Italy	Volunteers	Earthquake
Psychoeducational intervention to prevent critical incident stress among disaster volunteers*	Okanoya *et al*^41^	2014	Cross-sectional study	Japan	Volunteers	Earthquake, flood, typhoon, tsunami
Debriefing of American Red Cross personnel*	Armstrong^31^	1998	Cross-sectional study	USA	Professionals and volunteers	Earthquake
Therapeutic activism: Supporting emotional resilience of volunteers working in a refugee camp*	Hughes *et al*^60^	2019	Report	France	Volunteers	Displacement
A mental health program for ground zero rescue and recovery workers: Cases and observations*	Katz *et al*^67^	2006	Report	USA	Disaster workers, including volunteers	Terrorism
Preventing mental health risks in volunteers in disaster contexts: The case of the Villarrica Volcano eruption, Chile*	Espinoza *et al*^51^	2018	Mixed-methods study	Chile	Volunteers	Volcanic eruption
Organizational factors and mental health in community volunteers: The role of exposure, preparation, training, tasks assigned, and support*	Thormar *et al*^50^	2012	Cohort study	Indonesia	Volunteers	Earthquake
Supporting volunteer well-being through disaster: Perspectives and practices of a youth-led informal crisis volunteer group*	Nissen *et al*^68^	2022	Qualitative study	New Zealand	Volunteers	Earthquake, terrorism
A rapid realist review of group psychological first aid for humanitarian workers and volunteers*	Corey *et al*^35^	2021	Review paper	UK	Humanitarian workers, including volunteers	Unspecified
Integrative approach for the treatment of posttraumatic stress disorder in 9/11 first responders: Three core techniques*	Haugen *et al*^69^	2013	Concept note	USA	First responders, including volunteers	Terrorism
Implementation guide: Standards to facilitate the safety, security, and well-being of volunteers	IFRC^36^	2023	Guidelines		Volunteers	Unspecified
Guidelines for caring for staff and volunteers in crises	IFRC^37^	2019	Guidelines		Volunteers	Unspecified
Managing stress in humanitarian workers: Guidelines for good practice	Antares Foundation^43^	2012	Guidelines		Humanitarian workers, including volunteers	Unspecified
Caring for volunteers: A psychosocial support toolkit	IFRC^44^	2012	Toolkit		Volunteers	Unspecified
Volunteers in humanitarian settings	IFRC^45^	2022	Workshop material	Poland	Volunteers	Unspecified
Post traumatic stress disorder in first responders: The effectiveness of psychological intervention on stress response and coping styles	Arielle Hyler^33^	2010	Thesis paper	USA	Volunteer firefighters and transport union members	Fire incidents

* Peer-reviewed.

IFRC, International Federation of Red Cross and Red Crescent Societies.

### Humanitarian work-related

Two of the reviewed documents focused on volunteer firefighters working alongside civil firefighting groups.^32 33^ The rest involved volunteers who worked with various humanitarian organisations. The IFRC and its societies are mentioned in numerous documents. Other organisations, such as MSF, Médecins du Monde, Secours Catholique, and United Way, were also mentioned. The included literature documented different tasks performed by the volunteers in the field, as well as different PTEs that could trigger stress responses among the volunteers, giving rise to symptoms of trauma and/or acute stress. Table 2 lists these different tasks, PTEs, challenges, other factors and symptoms reported in the documents included.

**Table 2:** Lists of humanitarian tasks, PTEs, work-related challenges and risk factors and reported symptoms reported in the documents

Tasks	
Disaster response	Search and rescue, evacuation, debris removal, dead bodies or body parts removal, cutting steel, restoring infrastructure, firefighting
Medical and psychosocial support	Assisting the wounded, triage, psychological first aid
Non-medical support	Helping in refugee camps, running volunteer centres, aid and relief distribution
Potentially traumatic events (PTEs)	Primary and secondary exposure to violence, loss and suffering, seeing corpses and body parts, contact with victims with serious injuries, extended contact with traumatised victims, failed resuscitation attempts, doubts about the impact of their work, unrealistic expectations, physical difficulties or sickness, concerns about safety and security, risks of physical, mental and sexual assault, concerns about recurrence of disasters, gender inequalities, work-related challenges
Work-related challenges and risk factors	Harsh working conditions, physically difficult or dangerous tasks, moral or ethical dilemmas, lack of information, resources, preparation or support, internal conflicts, miscommunication, disagreements and disorganisation, heavy workload and long working hours, poor rest and sleep, lack of privacy and personal space, detachment from family, not being valued or acknowledged properly, police brutality
Reported symptoms	Hyperarousal, avoidance, flashbacks, intrusive memories, withdrawal, physical and mental exhaustion, apathy, self-destructive behaviours, psychosomatic complaints, low mood, agitation, mood difficulties, intense feelings, feeling inadequate, feeling guilt, burnout, emotional burden, emotional fatigue, reduced energy and efficiency, reduced motivation, pessimism, cynicism, psychosis, poor sleep, nightmares, alcohol consumption

### Interventions

The interventions discussed, suggested or evaluated in the reviewed documents can be broadly classified into 10 categories: psychoeducational, psychosocial, psychological first aid (PFA), psychological debriefing, therapy, counselling, preparatory training, screening and treatment, organised peer support system and multimodal approach. They aimed to alleviate acute stress, depression, anxiety and PTSD, as well as strengthen resilience, coping and psychosocial well-being. Most of the reviewed interventions were preventive in nature, comprising counselling sessions, educational sessions, debriefing, PFA, training, screening, etc. Only four interventions were mentioned to be therapeutic. Eleven documents out of twenty talked about intervening after the exposure, three were on pre-exposure interventions, two were on periexposure interventions and two were on multimodal interventions that supported the volunteers throughout their working period. Twelve of the documents discussed interventions that were already being implemented in the field of disaster response. The rest of them were either guidelines with the potential to be used in the field or possible interventions being evaluated through research.

For different interventions, the mode of application was different. Some were delivered by mental health professionals or clinicians, and some by trained staff or social workers. Both single and multiple individual and group sessions were conducted, fitting the respective contexts and interventions. Among the interventions identified by this scoping review, PFA was discussed more times than any other intervention, in four out of the 18 included documents.^34^,^37^ PFA is strongly advocated by the IFRC and widely implemented by its national societies. This intervention can be delivered by any person and involves actively listening to individuals seeking help, identifying any negative reactions and assisting them in addressing their immediate needs and problems by connecting them to the appropriate resources.^38^

Eight of the documents included were research papers that evaluated the effectiveness of their interventions of interest using different scale questionnaires or, in one case, a customised questionnaire. These scales served as screening tools, measuring subjective distress based on the number and severity of symptoms. A summary of these interventions has been provided as online supplemental material 3.

## Discussion

### Recent growth of literature

A total of 2627 documents were retrieved from electronic bibliographic databases and the archives of international organisations working with humanitarian assistance in disasters. After matching the eligibility criteria, only 20 documents were included in this scoping review, of which 14 were peer-reviewed and six were grey literature. This number is small considering that volunteers have been involved in humanitarian work worldwide for a long time. The first organised humanitarian volunteers date back to the late 19th century. In 1863, Henry Dunant, a Swiss businessman, founded the ICRC on witnessing the inhuman suffering of the soldiers from both sides on the battlefield of Solferino.^39^ The ICRC played an instrumental role in the development and promotion of International Humanitarian Law during armed conflicts, which is based on the 1949 Fourth Geneva Convention.^40^

The IFRC was formed in 1919 to address humanitarian needs in non-conflict settings. As mentioned earlier, it now has 191 national societies in different countries working with approximately 16 million local volunteers.^9 10^ Both organisations have heavily relied on local community volunteers for their operations in the field. Since then, other international and national humanitarian and/or volunteer organisations have emerged. Among the included documents, the earliest one on a PTSD intervention targeting humanitarian volunteers was a 1998 research paper that evaluated the effectiveness of multiple stressor debriefings on American Red Cross volunteers.^31^ The rest of the included documents date back to 2006 or later, suggesting that the issue has only recently gained further attention, giving momentum to discussions about it. However, it is also possible that research and data from earlier years were not available online.

### Mental health interventions

This scoping review identified several mental health interventions with the potential to prevent or relieve ill mental health symptoms in volunteers. Most of the interventions were implemented after deployment and subsequent exposure to PTEs. In an emergency situation following a disaster, first responders, including volunteers, must immediately dive into rescue and recovery work as an essential part of the first-line response, rarely getting adequate time to prepare and train for context-specific risks and challenges.^41 42^ It could explain why most of the interventions were implemented following response work. The reviewed literature supports both pre-exposure and postexposure interventions, with each phase offering unique benefits. No consensus was found that one timing could be better than the other. In this matter, the IFRC and the Antares Foundation have advocated a multimodal approach for the psychosocial well-being of their humanitarian volunteers.^43 44^ It encompasses measures taken throughout the full recruitment period for a volunteer, including post-deployment support. It starts with screening for mental health risk factors during recruitment and then progresses through preparatory training, organised peer support during deployment and monitoring, postdeployment PFA, reflection and appreciation and referral at the end of it all as required.^44 45^ A qualitative study by Umeda *et al* supported this method, recommending actions for each phase.^42^ Predeployment training could improve preparedness and resilience, and postdeployment interventions could address acute and ongoing psychological needs while also planning on how to manage on-site stress during the response work.^46 47^ While it may seem ideal and prudent to intervene at every step, many organisations may lack the time, money and other resources necessary. A brief session to prepare the volunteers for the mental health hazards and educate them to protect themselves could be a good place to start.^41^

Some of the interventions targeted specific mental health conditions, such as stress and depression, while others cared for the general psychological resilience and well-being of volunteers. PTSD, depression and anxiety are the most common concerns among first responders and emergency personnel.^14 48^ The question of whether to focus on addressing the most prevalent mental health issues or to design interventions that promote the overall mental well-being of volunteers remains open. This could be a valuable area for future research. Additionally, careful consideration is needed regarding the structure of interventions—specifically, when implementing individual sessions versus group sessions, to maximise their effectiveness. Predeployment group sessions arranged by respective organisations could prove to be essential in training and preparing the volunteers for exposures to PTEs; on the other hand, group debriefing sessions or similar activities in the presence of mental health professionals after the response work could potentially alleviate adverse mental health symptoms.^46 49^ Volunteers experiencing severe psychological distress would require personalised professional support in single sessions and/or referral to specialised care.

The IFRC had a direct or indirect link with many of the included documents. Four of the included documents were published by the IFRC (one workshop material, two guidelines and one toolkit)^36 37 44 45^ and three of the studies were done on Red Cross volunteers from three different national societies (American, Indonesian and Chilean Red Cross).^31 50 51^ The organisation appeared to have gone further than any other in developing and implementing psychosocial guidelines for humanitarian volunteers. Irrespective of the results, they have set an example in making efforts to protect the volunteers working with their national societies, which is commendable since mental health support for volunteers has so far relied on the goodwill of the organisations supervising them.

### Evaluation and challenges

A summary of different scales used to evaluate the reviewed interventions is provided as online supplemental material 4. It is worth noting that all the questionnaires were based on self-reported mental health symptoms. Individual perceptions could play a key role in this matter. Studies have shown that perceived social support, perceived threat and perceived needs are important factors in the development of PTSD.^52^,^54^ The same could be true for the effectiveness of other mental health interventions as well; whether the volunteers perceived the interventions to be useful or not could influence their effectiveness. However, only two of the studies asked the participants about their own perspectives regarding the interventions.^31 51^ The potential link between mental health interventions and individual perceptions of the volunteers is not clear and should be researched to observe its significance in evaluating the interventions.

There is no formal definition or job description for humanitarian volunteers that establishes the scope and limits of their work.^55^ As shown in table 2, the tasks vary depending on the needs of the respective contexts, and the risks that come with them vary accordingly, which could make it quite difficult to predict exposure to certain PTEs and address them. Moreover, it is difficult to determine which tasks would have a negative impact on the mental health of the volunteers. A study observed that volunteers who did not face severe experiences could also develop intense symptoms of acute stress.^41^ Seemingly harmless tasks that require close interaction with the trauma victims, such as providing PFA, could expose them to secondary trauma.^51 56^ Research has also shown that the cumulative effects of repeated exposure could lead to the development of PTSD, which is another factor to be considered.^57^ In the study by Armstrong *et al*, most of the stressful experiences reported by the volunteers were cumulative or chronic in nature.^31^ These issues could pose potential challenges when designing and evaluating mental health interventions targeting humanitarian volunteers.

### Missing information

A major portion of humanitarian volunteers are civilians who volunteer spontaneously and temporarily immediately after a disaster. These individuals, drawn mostly from the impacted community, offer their assistance as needed. According to the UNV, these local volunteers are the first line of response in the aftermath of disasters.^42^ However, no statistics could be found on the number or percentage of local volunteers among humanitarian workers. Also, this review could not find any document that specifically discussed this group of volunteers, which is why it is difficult to comment on their status.

There were no documents from the Middle East and North Africa (MENA) region or Sub-Saharan Africa, an important concern given that these two regions, so far, have experienced the highest number of armed conflicts in the 21st century, resulting in deaths and devastation.^58^ Another important yet missing region was South and Southwest Asia, where countries are at risk of losing more than 5% of their GDP each year due to disasters.^59^ In these regions, climate change effects combine with socioeconomic vulnerabilities and, in some cases, armed conflicts, resulting in detrimental impacts on people’s lives. In most cases, people are forced to migrate either within or outside the country, where they find refuge in camps supervised by the respective governments, local and international humanitarian organisations and their volunteers. Humanitarian organisations and volunteers play a vital role in helping people living in these camps overcome adversity by responding to their needs.

Only one of the included documents mentioned refugee camp volunteers in Calais, France, where displaced people had arrived from Sudan, Ethiopia, Eritrea and several other countries.^60 61^ Otherwise, no other document focused on volunteers working in camps. Due to their close interaction with internally displaced persons (IDPs) and refugees, volunteers working in refugee camps are at risk of experiencing secondary trauma.^62^ In fact, many of them were recruited from the affected communities.^63^ Additionally, they may also share the same safety and security concerns as the camp residents due to the political instability of the respective regions, as stated in the safety and security guidelines for humanitarian volunteers developed by the ICRC.^24 64^ Given these factors, volunteers operating in these regions should be at risk of adverse mental health effects, which is why it is also important to study the contexts and mental health conditions of humanitarian volunteers in MENA and sub-Saharan Africa.

### Methodological strengths and limitations

The flexible methodology of scoping review was suitable to conduct a comprehensive search across a heterogeneous body of literature and diverse information sources, such as electronic databases and organisational websites. It allowed us to explore the extent and nature of all existing evidence, including both peer-reviewed and grey literature, and summarise the results to have a better understanding of the matter. The clearly defined objectives, eligibility criteria and overall methodology contributed to the transparency of the research. The review adhered to the established guidelines of PRISMA-ScR to systematically conduct research and report the findings. Thus, it helped identify the gaps in knowledge and indicate the areas where further research is needed. The findings also determined the feasibility of undertaking a systematic review of the same topic.

In a scoping review, there is always a chance of bias due to the degree of subjectivity in the process of selecting the articles and the lack of a robust search strategy in the case of grey literature. Moreover, a scoping review does not include a detailed critical appraisal of the quality of the included documents, which puts the methodological rigour of the studies and the reliability of the findings in question. Another limitation of this study is that it does not discuss the individual components of the reviewed interventions, which could be insightful. The heterogeneity of the interventions in question made it difficult to discuss and compare their components individually. Moreover, as this study focused on how they were evaluated rather than how effective they were, it focused more on the general characteristics. Lastly, because of the narrative style of evidence synthesis, no statistical inference could be made in this review.

## Conclusions and recommendations

Over the last two decades, evidence on PTSD interventions for humanitarian volunteers has grown, suggesting an increasing awareness of volunteers’ psychosocial well-being. This review has identified distinct types of interventions that are either in place or suggested by researchers and experts. Additionally, there are guidelines outlining standard practices for caring for volunteers’ mental health. However, the diversity of tasks, the unpredictability of exposure to PTEs and the unique characteristics of volunteers complicate the understanding of their mental health needs. Qualitative research on the lived experiences of volunteers could lead the way in this matter. Although some preventive and therapeutic interventions show promise, their effectiveness and generalisability require further investigation. The studies evaluating the effectiveness of potential interventions were heterogeneous in terms of their settings, study designs, interventions evaluated and evaluation methods used, dismissing the possibility of a systematic review on the topic. Lastly, the lack of research on volunteers in high-risk regions like MENA and sub-Saharan Africa is a significant gap.

To enhance the mental health support for humanitarian volunteers, it is crucial to generate scientific evidence, particularly in high-risk regions. It is also important to include a broader range of volunteers in research, especially local and spontaneous responders. Future studies should aim for more rigorous methodological approaches and consider the volunteers’ perspectives on the interventions. We need to compare different interventions to determine which ones are more effective for humanitarian volunteers. The intervention schedule and cost-effectiveness are also important concerns. We recommend comparing pre-exposure and postexposure interventions with multimodal systems. Furthermore, the development and implementation of cost-effective, context-specific interventions should be prioritised. Lastly, organisational policies need to incorporate the existing mental healthcare guidelines for volunteers to ensure their psychosocial well-being.

## Supplementary material

10.1136/bmjopen-2024-095363 online supplemental file 1

10.1136/bmjopen-2024-095363 online supplemental file 2

10.1136/bmjopen-2024-095363 online supplemental file 3

10.1136/bmjopen-2024-095363 online supplemental file 4

## Data Availability

Data are available upon reasonable request.
