# Supplementary material for: Mental health interventions for humanitarian volunteers: a scoping review
Source: BMJ Open. 2025 Jul 6;15(7):e095363. doi: 10.1136/bmjopen-2024-095363 (PMC12230954; doi:10.1136/bmjopen-2024-095363)
Supplement: online supplemental file 2 [file bmjopen-15-7-s002.docx]

**Search queries for different academic databases**

**Initial search queries**

| **PubMed** | | |
| --- | --- | --- |
| **Search** | **Query** | **Results** |
| #1 | **(((((((("stress disorders, post traumatic"[MeSH Terms]) OR ("compassion fatigue"[MeSH Terms])) OR (post traumatic stress disorder*)) OR (post traumatic neurosis)) OR (PTSD)) OR (secondary traumatic stress)) OR (secondary traumatization)) OR (vicarious trauma*)) OR (vicarious traumatization)** | 63,236 |
| #2 | ((((((((((("psychosocial intervention"[MeSH Terms]) OR (intervention*)) OR (therap*)) OR (counsel*)) OR ("psychological first aid")) OR ("psychological debriefing")) OR (psychosocial intervention*)) OR (psychological intervention*)) OR (support*)) OR (training*)) OR (program*)) OR (screening) | 21,550,865 |
| #3 | (((((((("volunteers"[MeSH Terms]) OR (volunteer*)) OR (humanitarian volunteer*)) OR (community volunteer*)) OR (voluntary worker*)) OR (voluntary rescuer*)) OR (voluntary responder*)) OR (voluntary rescue worker*)) OR (voluntary first responder*) | 250,281 |
| #4 | ((((((((((((("disasters"[MeSH Terms]) OR ("armed conflicts"[MeSH Terms])) OR ("accidents"[MeSH Terms])) OR ("terrorism"[MeSH Terms])) OR (disaster*)) OR (war*)) OR (terrorism)) OR (accident*)) OR (natural disaster*)) OR (armed conflict*)) OR (catastrophe*)) OR (cris*s)) OR (emergenc*)) OR (calamit*) | 1,161,241 |
| #5 | #1 AND #2 AND #3 AND #4 | 203 |
|  | MeSH = Medical subject headings |  |
| **Embase** | | |
| **Search** | **Query** | **Results** |
| #1 | 'posttraumatic stress disorder'/exp OR 'posttraumatic stress disorder' OR 'vicarious trauma'/exp OR 'vicarious trauma' OR 'secondary traumatic stress'/exp OR 'secondary traumatic stress' OR 'ptsd'/exp OR 'ptsd' OR 'posttraumatic neurosis'/exp OR 'posttraumatic neurosis' OR 'secondary traumatization'/exp OR 'secondary traumatization' OR 'vicarious traumatization'/exp OR 'vicarious traumatization' OR 'compassion fatigue'/exp OR 'compassion fatigue' | 95,120 |
| #2 | 'psychosocial intervention'/exp OR 'psychosocial intervention' OR 'psychotherapy'/exp OR 'psychotherapy' OR 'psychosocial care'/exp OR 'psychosocial care' OR 'psychological counseling'/exp OR 'psychological counseling' OR 'psychological first aid'/exp OR 'psychological first aid' OR 'psychological debriefing'/exp OR 'psychological debriefing' OR 'intervention*' OR 'therap*' OR 'counseling'/exp OR 'counseling' OR 'support*' OR 'training*' OR 'program*' | 16,766,485 |
| #3 | 'volunteer'/exp OR 'volunteer' OR 'voluntary worker'/exp OR 'voluntary worker' OR 'humanitarian volunteer*' OR 'community volunteer*' OR 'voluntary rescuer*' OR 'voluntary responder*' OR 'voluntary first responder*' OR 'voluntary emergency responder*' OR 'voluntary relief worker*' OR 'voluntary rescue worker*' | 102,913 |
| #4 | 'disaster'/exp OR 'disaster' OR 'war'/exp OR 'war' OR 'terrorism'/exp OR 'terrorism' OR 'accident'/exp OR 'accident' OR 'natural disaster*' OR 'catastrophe*' OR 'cris*s' OR 'emergenc*' OR 'calamit*' | 1,990,966 |
| **#**5 | #1 AND #2 AND #3 AND #4 | 252 |
|  | exp = explode |  |
| **Web of Science** | | |
| **Search** | **Query** | **Results** |
| #1 | (((((((TS=(post traumatic stress disorder)) OR TS=(PTSD)) OR TS=(post traumatic neurosis)) OR TS=(secondary traumatic stress)) OR TS=(secondary traumatization)) OR TS=(vicarious trauma)) OR TS=(vicarious traumatization)) OR TS=(compassion fatigue) | 61,990 |
| #2 | (((((((((((TS=(intervention*)) OR TS=(psychosocial intervention*)) OR TS=(psychological first aid)) OR TS=(psychological debriefing)) OR TS=(psychotherap*)) OR TS=(therap*)) OR TS=(counsel*)) OR TS=(support*))) OR TS=(screening)) OR TS=(training*)) OR TS=(program*) | 10,956,396 |
| #3 | (((((((((TS=(volunteer*)) OR TS=(humanitarian volunteer*)) OR TS=(community volunteer*)) OR TS=(voluntary responder*)) OR TS=(first responder*)) OR TS=(voluntary first responder*)) OR TS=(voluntary worker*)) OR TS=(voluntary rescuer*)) OR TS=(voluntary rescue worker*)) OR TS=(voluntary relief worker*) | 245,865 |
| #4 | ((((((((TS=(disaster*)) OR TS=(natural disaster*)) OR TS=(war*)) OR TS=(armed conflict*)) OR TS=(terrorism)) OR TS=(accident*)) OR TS=(catastrophe*)) OR TS=(cris*s)) OR TS=(emergenc*) OR TS=(calamit*) | 2,381,565 |
| **#**5 | #1 AND #2 AND #3 AND #4 | 299 |
|  | TS = Topic |  |
| **EBSCOhost (PsycINFO & CINAHL)** | | |
| **Search** | **Query** | **Results** |
| #1 | SU (post traumatic stress disorder or ptsd or posttraumatic stress disorder or post-traumatic stress disorder) OR SU (secondary traumatic stress or compassion fatigue or vicarious trauma) OR TX post traumatic stress disorder OR TX PTSD | 175,702 |
| #2 | SU psychosocial interventions OR SU psychotherapy OR SU psychological first aid OR SU psychological debriefing OR SU psychological counseling OR SU psychosocial support OR TX intervention* OR TX training* OR TX program* OR TX therap* OR TX counsel* OR TX support* | 25,418,930 |
| #3 | SU (volunteering or volunteer or volunteerism) OR TX community volunteer* OR TX humanitarian volunteer* OR TX voluntary worker* OR TX voluntary responder* OR TX voluntary rescuer* | 86,697 |
| #4 | SU natural disasters OR SU (armed conflict or war) OR SU terrorism OR SU accidents OR TX disaster* OR TX catastrophe* OR TX cris*s OR TX emergenc* OR TX calamit* | 1,839,184 |
| #5 | #1 AND #2 AND #3 AND #4 | 494 |
|  | SU = Subject terms; TX = All text |  |
| **Total 1,248** | | |

**Updated search queries**

| **PubMed** | | |
| --- | --- | --- |
| **Search** | **Query** | **Results** |
| #1 | (((((("mental health"[MeSH Terms]) OR ("mental health")) OR ("mental hygiene")) OR ("psychological well-being")) OR (“psychological resilience”)) OR (“well-being”)) OR (“resilience”) | 599,743 |
| #2 | ((((((((("mental health services"[MeSH Terms]) OR ("psychosocial intervention"[MeSH Terms])) OR ("psychotherapy"[MeSH Terms])) OR (interven*)) OR (therap*)) OR (psychotherap*)) OR (counsel*)) OR (program*)) OR (support*)) OR (train*) | 19,747,218 |
| #3 | (((((((("volunteers"[MeSH Terms]) OR (volunteer*)) OR (humanitarian volunteer*)) OR (community volunteer*)) OR (voluntary worker*)) OR (voluntary rescuer*)) OR (voluntary responder*)) OR (voluntary rescue worker*)) OR (voluntary first responder*) | 259,740 |
| #4 | ((((((((((((("disasters"[MeSH Terms]) OR ("armed conflicts"[MeSH Terms])) OR ("accidents"[MeSH Terms])) OR ("terrorism"[MeSH Terms])) OR (disaster*)) OR (war*)) OR (terrorism)) OR (accident*)) OR (natural disaster*)) OR (armed conflict*)) OR (catastrophe*)) OR (cris*s)) OR (emergenc*)) OR (calamit*) | 1,366,136 |
| #5 | #1 AND #2 AND #3 AND #4 | 666 |
|  | MeSH = Medical subject headings |  |
| **Embase** | | |
| **Search** | **Query** | **Results** |
| #1 | 'mental health' OR 'psychological resilience' OR 'psychological well-being' OR 'mental hygiene' OR 'wellbeing' OR 'resilience' | 991,972 |
| #2 | 'psychosocial intervention'/exp OR 'psychosocial intervention' OR 'psychotherapy'/exp OR 'psychotherapy' OR 'psychosocial care'/exp OR 'psychosocial care' OR 'psychological counseling'/exp OR 'psychological counseling' OR 'psychological first aid'/exp OR 'psychological first aid' OR 'psychological debriefing'/exp OR 'psychological debriefing' OR 'intervention*' OR 'counseling'/exp OR 'counseling' OR 'support*' OR 'train*' OR 'program*' OR 'therap*' | 18,039,550 |
| #3 | 'volunteer'/exp OR 'volunteer' OR 'voluntary worker'/exp OR 'voluntary worker' OR 'humanitarian volunteer*' OR 'community volunteer*' OR 'voluntary rescuer*' OR 'voluntary responder*' OR 'voluntary first responder*' OR 'voluntary emergency responder*' OR 'voluntary relief worker*' OR 'voluntary rescue worker*' | 100,960 |
| #4 | 'disaster'/exp OR 'disaster' OR 'war'/exp OR 'war' OR 'terrorism'/exp OR 'terrorism' OR 'accident'/exp OR 'accident' OR 'natural disaster*' OR 'catastroph*' OR 'cris*s' OR 'emergenc*' OR 'calamit*' | 2,179,282 |
| **#**5 | #1 AND #2 AND #3 AND #4 | 513 |
|  | exp = explode |  |
| **Web of Science** | | |
| **Search** | **Query** | **Results** |
| #1 | ((((TS=(“mental health”)) OR TS=(“mental hygiene”)) OR TS=(“psychological well-being”)) OR TS=(“psychological resilience”)) OR TS=(“resilience”)) OR TS=(“well-being”) | 604,500 |
| #2 | (((((((((((TS=(interven*)) OR TS=(therap*)) OR TS=(psychotherapy*)) OR TS=(counsel*)) OR TS=(support*)) OR TS=(train*)) OR TS=(program*)) OR TS=(“mental health service”))) OR TS=(“psychosocial intervention”)) OR TS=(“psychological debriefing”)) OR TS=(“psychological first aid”) | 12,263,850 |
| #3 | (((((((((TS=(volunteer*)) OR TS=(humanitarian volunteer*)) OR TS=(community volunteer*)) OR TS=(voluntary responder*)) OR TS=(first responder*)) OR TS=(voluntary first responder*)) OR TS=(voluntary worker*)) OR TS=(voluntary rescuer*)) OR TS=(voluntary rescue worker*)) OR TS=(voluntary relief worker*) | 289,159 |
| #4 | ((((((((TS=(disaster*)) OR TS=(natural disaster*)) OR TS=(war*)) OR TS=(armed conflict*)) OR TS=(terrorism)) OR TS=(accident*)) OR TS=(catastrophe*)) OR TS=(cris*s)) OR TS=(emergenc*) OR TS=(calamit*) | 2,878,371 |
| **#**5 | #1 AND #2 AND #3 AND #4 | 924 |
|  | TS = Topic |  |
| **EBSCOhost (Medline, PsycINFO & CINAHL)** | | |
| **Search** | **Query** | **Results** |
| #1 | SU mental health OR SU mental hygiene OR SU psychological well-being OR SU mental well-being OR psychological resilience OR TX mental health OR TX resilience OR TX well-being | 2,785,435 |
| #2 | SU mental health services OR SU psychosocial interventions OR SU psychotherapy OR SU psychological first aid OR SU psychological debriefing OR SU psychological counseling OR SU psychosocial support OR TX interven* OR TX train* OR TX program* OR TX therap* OR TX counsel* OR TX support* OR TX psychotherap* | 37,255,551 |
| #3 | SU (volunteering or volunteer or volunteerism) OR TX community volunteer* OR TX humanitarian volunteer* OR TX voluntary worker* OR TX voluntary responder* OR TX voluntary rescuer* OR TX emergency responder* OR TX emergency rescuer* OR TX relief worker* | 136,471 |
| #4 | SU natural disasters OR SU (armed conflict or war) OR SU terrorism OR SU accidents OR TX disaster* OR TX catastroph* OR TX cris*s OR TX emergenc* OR TX calamit* OR TX accident* | 5,749,586 |
| #5 | #1 AND #2 AND #3 AND #4 | 506 |
|  | SU = Subject terms; TX = All text  Filters applied = full text, peer-reviewed, academic journals |  |
| **Total 2,609** | | |
