# Supplementary material for: Mental health interventions for humanitarian volunteers: a scoping review
Source: BMJ Open. 2025 Jul 6;15(7):e095363. doi: 10.1136/bmjopen-2024-095363 (PMC12230954; doi:10.1136/bmjopen-2024-095363)
Supplement: online supplemental file 3 [file bmjopen-15-7-s003.docx]

**Summary of the reviewed interventions**

| **Intervention schedule** | **Author / Organization** | **Intervention** | **Outcome of interest** | **Implemented in the field** | **Mode of application** | **Evaluation method** | **Conclusions / Remarks/ Recommendations** |
| --- | --- | --- | --- | --- | --- | --- | --- |
| Pre-exposure | Okanoya et al. (38) | Psychoeducational intervention (PEI) | Critical incident stress (CIS) | No | Single group session with regular staff or researchers | Comparison between volunteers with and without PEI using IES-R* | PEI before deployment was significantly related to alleviated CIS in volunteers who received it |
| Pre-exposure | Scuri et al. (37) | Resilience training | Resilience | Yes | Group sessions arranged by the affiliated organizations | Differences in coping and resilience measured by paired t-tests using CISS* and RS* | Medium to high levels of coping and resilience were linked to pre-departure training and preparation |
| Pre-exposure | Thormar et al. (36) | Organizational support, preparation and training | PTSD, depression, and anxiety | Yes | Routine organizational training and preparation through providing adequate information and necessary equipment along with organizational mental and technical support | Association between organizational factors and mental health symptoms were measured using IES-R*, HADS*, SHC inventory scoring system, and TESS* | Training and preparation on personal safety and the need for support had a significant effect on both PTSD and anxiety. Anxiety was also linked with the information and equipment received before deployment. |
| Post-exposure | Armstrong et al. (31) | Multiple Stressor Debriefing (MSD) | Stress | Yes | Single individual or group sessions with a clinical psychologist, psychiatrist, or social worker | Comparison between individual and group debriefing participants using a custom debriefing questionnaire | The volunteers evaluated both single and group debriefings positively. They were more likely to feel like they had expressed their feelings when there were fewer participants per group. |
| Post-exposure | Bekircan et al. (33) | Psychological first aid (PFA) | Stress and resilience | Yes | Multiple online sessions with a trained mental health nurse in groups | Pre-test and post-test comparison between volunteers with and without PFA using STSS* and BRS* | The level of stress was observed to be considerably lower in the group receiving PFA |
| Post-exposure | Corey et al. (41) | Group psychological first aid (PFA) | Stress | Yes | Group sessions with mental health professionals, trained workers, volunteers, or peers |  | GPFA helps the humanitarian workers to understand their natural reactions, develop adaptive coping strategies, and build social connections, promoting a sense of belonging and security |
| Post-exposure | Espinoza et al. (40) | "Emotional Containment with Emergency Volunteers" psychosocial intervention with art therapy | Stress and emotional exhaustion | No | Single art therapy and interview sessions with psychologists in a group |  | Psychosocial interventions are important both during and after the disaster response. Art therapy helped to increase self-awareness of stress and emotional exhaustion |
| Post-exposure | Haugen et al. (44) | Integrative psychotherapy | PTSD | No | Multiple individual sessions with clinicians |  | Psychotherapy was believed to yield positive treatment outcomes in participants with full or partial PTSD |
| Post-exposure | Katz et al. (43) | Screening and treatment through a mental health program | Mental health needs | Yes | Individual sessions with clinicians, psychiatrists, or social workers |  | The program reveals the emotional toll on rescue and recovery workers and their diverse mental health needs after the World Trade Center (WTC) tragedy, providing valuable lessons |
| Post-exposure | Mahaffey et al. (34) | Disaster Worker Resiliency Training Program (DWRT) | PTSD, depression, and perceived stress | No | Individual and group sessions with mental health professionals | Baseline survey and follow up after 3 months to compare volunteers with and without DWRT using PCL-5*, PHQ-9*, and PSS* | Reduced incidence of mental health symptoms in volunteers who received DWRT |
| Post-exposure | Müller et al. (32) | Digital mindfulness meditation | Mental health | No | One group received an app-based mindfulness intervention for 6 weeks, followed by the control group receiving the same for the following 6 weeks | Data on different aspects of mental health were collected during baseline, after 6 weeks and after 12 weeks using custom questionnaires | Digital mindfulness effectively improved the mental health of the participants with significant interaction effects of time and intervention for all variables |
| Post-exposure | Tuckey et al. (35) | Group critical incident stress debriefing (CISD) | PTSD | Yes | Single group session with mental health professionals | Pre- and post-intervention results comparing CISD with screening and education using IES-R*, K10*, and quality of life questionnaire | CISD had no significant effects on post-traumatic stress or distress, and an occupational health approach was suggested |
| Post-exposure | IFRC (45) | Psychological first aid (PFA) and referrals | Psychosocial well-being | Yes | Individual and group sessions of PFA by the organization |  | Attentions must also be paid to internal risks and threats to mental health |
| Post-exposure | Arielle Hyler (50) | Critical incident stress management (CISM) and counseling | Critical incident stress (CIS) | Yes | Group and individual sessions arranged by the organization | Comparison was made between volunteers receiving CISM and transport union members using IES-R*, PHQ-9*, and brief COPE inventory | There was no significant difference between the two groups in stress response, but those who received the intervention showed more positive coping styles |
| Peri-exposure | Hughes et al. (42) | Therapeutic activism | Resilience | Yes | Weekly individual and group sessions with psychotherapists and psychologists |  | Emotional support for volunteers working with refugees was crucial, helping them to bring productive and life-enhancing changes |
| Peri-exposure | IFRC (49) | Organized peer support system | PTSD, distress, depression, anxiety | Not mentioned | Informal defusing by peers in everyday context |  | Single interventions do not have significant effects on mental health. Organized systems that include prevention, preparation, mission support, and aftercare have positive effects. |
| Peri- and post-exposure | IFRC (46) | Psychological first aid (PFA) and referrals | Psychosocial well-being | Yes | Individual and group sessions of PFA and psychoeducation by the organization |  | Providing psychosocial support to volunteers is essential to care for their well-being and often leads to increased recruitment and retention |
| Peri- and post-disaster | Nissen et al. (39) | Multimodal | Psychosocial well-being | Yes | Three interrelated practices termed as “action”, “reflection” and “connection” |  | Organizations may adopt similar practices or benefit from external support. Volunteers can continue to reflect on their engagement long after the disaster phase, pointing to the value of longitudinal perspectives. |
| Pre-, peri- and post-exposure | IFRC (48) | Multimodal | Psychosocial well-being | Not mentioned | Screening during recruitment, preparation and training, contingency planning, monitoring, team meetings, peer support, reflection appreciation, referral, and psychological first aid (PFA) |  | It is obligatory to support the well-being of volunteers before, during and after the emergency response work |
| Pre-, peri- and post-exposure | Antares Foundation (47) | Multimodal | Stress | Not mentioned | Policy making, screening, training, monitoring and support by the organization |  | Humanitarian workers, including volunteers, are exposed to a wide variety of sources of stress. Good psychosocial care is an important asset in the prevention, management, and treatment of stress. |

**IES-R: impact of event scale-revised; STSS: secondary traumatic stress scale; BRS: brief resilience scale; PCL-5: PTSD checklist for DSM 5; PHQ-9: patient health questionnaire-9; PSS: perceived stress scale; CISS: coping inventory for stressful situation; RS: resilience scale; K10: Kessler-10; HADS: hospital anxiety and depression scale; TESS: traumatic exposure severity scale*
