# Supplementary material for: Mental health interventions for humanitarian volunteers: a scoping review
Source: BMJ Open. 2025 Jul 6;15(7):e095363. doi: 10.1136/bmjopen-2024-095363 (PMC12230954; doi:10.1136/bmjopen-2024-095363)
Supplement: online supplemental file 4 [file bmjopen-15-7-s004.docx]

**Summary of different scales used to evaluate the interventions**

| **Ref** | **Scale*** | **Use** | **Target Population** | **Items** | **Time Frame** |
| --- | --- | --- | --- | --- | --- |
| (1) | IES-R  (Impact of Event Scale-Revised) | Monitors PTSD symptoms | Adolescents, adults, & older adults | 22 | Past 1 week |
| (2) | STSS  (Secondary Traumatic Stress Scale) | Monitors secondary traumatic stress symptoms | Adults, especially people exposed to others' trauma | 17 | Past 1 week |
| (3) | BRS  (Brief Resilience Scale) | Assesses the ability to recover from stress | Late adolescents, adults, & older adults | 6 | Not specified |
| (4) | PCL-5  (PTSD Checklist for DSM-5) | Monitors PTSD symptoms | Adults, sometimes late adolescents | 20 | Past 1 week or 1 month |
| (5) | PHQ-9  (Patient Health Questionnaire-9) | Screening, diagnosing, monitoring and measuring the severity of depression | Adolescents, adults, & older adults | 9 | Past 2 weeks |
| (6) | PSS-10  (Perceived Stress Scale) | Measures perceived stress levels | Adolescents, adults, & older adults | 10 | Past 1 month |
| (7) | CISS  (Coping Inventory for Stressful Situations) | Assesses coping strategies | Adolescents, adults, & older adults | 48 | Not specified |
| (8) | RS  (Resilience Scale) | Assesses the ability to recover from stress | Initially developed for older women, but now widely validated in adolescents, adults & older adults | 25 | Not specified |
| (9) | K10  (Kessler Psychological Distress Scale) | Measures psychological distress | Adolescents, adults, & older adults | 10 | Past 1 month |
| (10) | HADS  (Hospital Anxiety and Depression Scale) | Assesses anxiety and depression | Adults & older adults in hospital settings | 14 | Past 1 week |
| (11) | TESS  (Traumatic Exposure Severity Scale) | Assesses the severity of exposure to trauma | Adults & sometimes adolescents, especially disaster survivors | 24 | Not specified |

**All the scales are based on self-reported symptoms*

**References**

1. Weiss DS. The Impact of Event Scale: Revised. In: Wilson JP, Tang CS kum, editors. Cross-Cultural Assessment of Psychological Trauma and PTSD [Internet]. Boston, MA: Springer US; 2007 [cited 2025 May 27]. p. 219–38. (International and Cultural Psychology Series). Available from: http://link.springer.com/10.1007/978-0-387-70990-1_10

2. Bride BE, Robinson MM, Yegidis B, Figley CR. Development and Validation of the Secondary Traumatic Stress Scale. Res Soc Work Pract. 2004 Jan;14(1):27–35.

3. Smith BW, Dalen J, Wiggins K, Tooley E, Christopher P, Bernard J. The brief resilience scale: Assessing the ability to bounce back. Int J Behav Med. 2008 Sep;15(3):194–200.

4. Blevins CA, Weathers FW, Davis MT, Witte TK, Domino JL. The Posttraumatic Stress Disorder Checklist for DSM-5 (PCL-5): Development and Initial Psychometric Evaluation. J Trauma Stress. 2015 Dec;28(6):489–98.

5. Kroenke K, Spitzer RL, Williams JBW. The PHQ-9: Validity of a brief depression severity measure. J Gen Intern Med. 2001 Sep;16(9):606–13.

6. Cohen S, Kamarck T, Mermelstein R. A Global Measure of Perceived Stress. J Health Soc Behav. 1983;24(4):385–96.

7. Endler NS, Parker JDA. Assessment of multidimensional coping: Task, emotion, and avoidance strategies. Psychol Assess. 1994 Mar;6(1):50–60.

8. Wagnild GM, Young HM. Development and psychometric evaluation of the Resilience Scale. J Nurs Meas. 1993;1(2):165–78.

9. Kessler RC, Andrews G, Colpe LJ, Hiripi E, Mroczek DK, Normand SLT, et al. Short screening scales to monitor population prevalences and trends in non-specific psychological distress. Psychol Med. 2002 Aug;32(6):959–76.

10. Zigmond AS, Snaith RP. The Hospital Anxiety and Depression Scale. Acta Psychiatr Scand. 1983 Jun;67(6):361–70.

11. Elal G, Slade P. Traumatic Exposure Severity Scale (TESS): A measure of exposure to major disasters. J Trauma Stress. 2005 Jun;18(3):213–20.
