# Supplementary material for: Mental health interventions for humanitarian volunteers: a scoping review
Source: BMJ Open. 2025 Jul 6;15(7):e095363. doi: 10.1136/bmjopen-2024-095363 (PMC12230954; doi:10.1136/bmjopen-2024-095363)
Supplement: Supplementary file 5 [file bmjopen-2024-095363.reviewer_comments.pdf]

PEER REVIEW HISTORY

BMJ Open publishes all reviews undertaken for accepted manuscripts. Reviewers are asked to complete a checklist review form and are provided with free text boxes to elaborate on their assessment. These free text comments are reproduced below.

ARTICLE DETAILS

Title (Provisional)

Mental health interventions for humanitarian volunteers: a scoping review

Authors

Nasrullah, Sarker Mohammad; Refat, Tanjamul; Gustavsson, Martina Emelia

---

VERSION 1 - REVIEW

**Reviewer**	**1**
**Name**	**Aldamman, Kinan**
**Affiliation**	**International Committee of the Red Cross**
**Date**	**27-Mar-2025**
**COI**	**None**

I would like to thank the authors for preparing this interesting scoping review and would like to raise some potential points for their consideration to improve the manuscript:

1. The consistency with some psychotraumatic terminologies may benefit from a revision. For example, P3 L18 “developing stress”, P7 L20 “acute stress”, P8 L9 “Early interventions to address mental well-being or acute stress”, P14 L14 “could trigger stress”. It is advised to be consistent with the concepts of acute stress reaction (ICD), disorder (DSM), or using the term stress response (when describing the normal physiological phenomenon).

2. To improve the readability, it is advised to add an additional column in Table 1 P13, of either the title or the study hypothesis for each of the included articles instead of keeping this information available only in the supplement materials.

3. References number 53, 54, and 55 don’t focus on PTSD per se yet they are included. This is a call for authors to either revise their inclusion/exclusion criteria or limit those with sole focus on PTSD to peer-reviewed articles. Later in their review, the author states similar conclusions “The outcome of interest was not always PTSD. In some cases, these interventions aimed to alleviate stress, depression, and anxiety, as well as strengthen resilience, coping, and psychosocial well-being, which would in turn help to prevent PTSD”.

4. The authors are advised to revise their extractions to match the exact conclusions of the included articles. For example, Thormar et al., in Table P 38 L 31 (is not actually reference number 30 but number 24!), doesn't include group sessions for preparation. Furthermore, it concludes that there is no association between PTSD and training, but only with safety, referencing the regression table in that article. Thormar et al.'s study hasn't investigated the difference between those who were trained and those who were not. In conclusion, that study has no sufficient assumptions to make any interference of training as an intervention. "Although we expected the more experienced volunteers to have lower symptoms, this was not borne out in the regression analysis. The same applied to those who felt less trained. However, preparation in terms of good information about the situation, quality of equipment to be used, and proper safety measures in place were important," citing that article itself.

5. The section "Factors to be considered P16 L40" doesn't clearly serve the purpose of the review. I encourage the authors to revise the links between these factors and the interventions for PTSD for volunteers if they decide to keep it. The personality trait screening proposal looks discriminatory and out of context in humanitarian settings. Authors are requested to bring evidence to support it if they recommend that for humanitarian organisations. Based on the above comment, it might make more sense to discuss this section after the interventions themselves, making some recommendations for practice.

6. In P18 L30, the article argues that "treating PTSD is quite tedious and lengthy, often not ending with satisfactory results unless intervention is made at an early stage". The authors don't bring up-to-date evidence in support of this statement. The diagnosis of PTSD cannot be made before 1 month of exposure (ICD-11), and there are quite decent discrepancies in the effectiveness of early interventions in the literature. The reference provided is from 25 years ago, and the other one only looked at early interventions to prevent, not at the treatment of PTSD!

7. In the nature of interventions in P18, the authors argue that most organisations would prefer post-exposure interventions due to a lack of time and resources in preparing volunteers. I am unaware of any even anecdotal discussion favouring post-exposure intervention over the preparedness of volunteers for resource reasons as these interventions are also costly and require considerable levels of coordination and follow-up. Most probably this interference is attributable to publication bias, as it is difficult to evaluate the preparedness programmes, Thormar et al.'s article is a good example of such. The authors are encouraged to bring evidence supporting their argument.

8. In their argument around the nature of interventions in P18, the authors may also wish to consider bringing ethical and duty-of-care perspectives to complement their argument, not only to point out the cost-effectiveness part.

9. Contrary to the inclusion criteria of this review, the PFA approach discussed in P18 and 18 doesn't aim to prevent PTSD, but rather aims to address the common mental health conditions and wellbeing after exposure to PTEs.

10. In evaluating the interventions, the article misses the opportunity to discuss the components of these interventions rather than listing them, and what makes them special for volunteers. There is little discriminatory explanation of, for example, if PFA or CISM are different for volunteers over paid staff or not, what components of preparation for the work are recommended, who provides the interventions and at what time from the exposure and does each of these interventions include and how do they differ from each other… etc. The authors are encouraged to bring these aspects into their discussion.

11. In the same section "evaluating the interventions", the part on DSM, rather than ICD criteria looks out of the scope of the study as this review doesn't compare the discrepancy of diagnostic tools for PTSD. Most of the included studies compare the symptoms, in terms of scores pre- and post-intervention rather than prevalences, in terms of cut-offs. Unless the authors bring examples of the diagnostic in PTSD (in terms of prevalence % before and after intervention), this argument could be made redundant.

12. In P 19 L2, the authors bring an argument about the perception of volunteers around the intervention as a potential contributor to the actual effectiveness. Even when 2 of the included articles had addressed this matter, there is no explanation of how so. Authors are encouraged to elaborate on this matter in light of included articles.

13. The paper would benefit from a summary table of these inventions before, peri exposure and after exposure interventions.

14. After reading the whole manuscript, it seems like it looks at interventions to address the adverse mental health consequences of PTEs rather than PTSD among humanitarian volunteers. The outcomes of the included articles, particularly with prevention, go beyond PTSD per se. The authors are encouraged to refine their aim statement accordingly.

**Reviewer**	**2**
**Name**	**Edgar, Nicole**
**Affiliation Program**	**Ottawa Hospital Research Institute, Clinical Epidemiology**
**Date**	**07-Apr-2025**
**COI**	**None**

Thank you for the opportunity to review this scoping review entitled “Post-traumatic stress disorder interventions for humanitarian volunteers: a scoping review”. However , I feel that major revisions are required to consider this for publication. Once revisions are complete, I feel that this manuscript will fill a gap on understanding what interventions for PTSD are available for humanitarian volunteers.

I have some overarching concerns which i feel need to be addressed:

1. The manuscript does not seem to focus on humanitarian volunteers or specifically PTSD interventions.

a. The Summary of Interventions supplementary table indicates that 7/12 of the interventions identified were not available to volunteers. How did these papers meet eligibility criteria to be included? I am not sure that these can be considered “interventions for humanitarian volunteers” if half the interventions were not available to volunteers. The authors should clarify or revise.

b. In the results, the authors state that the outcome of interest “was not always PTSD”, however their inclusion criteria state that “documents that explored any other health condition beyond or unrelated to PTSD would be excluded”. This should be clarified further. The authors state that these other outcomes “would in turn help to prevent PTSD”, however I am not sure what evidence is provided to support this. As the author’s stated aim is to describe PTSD interventions, the results should either: be focused on PTSD specifically or more clearly draw the link between the non-PTSD outcomes and how this impacts PTSD.

3. The results do not highlight what types of interventions were identified or provide any sort of narrative that addresses their research question. The authors note that this is in the “supplemental material” but a high-level overview of the interventions identified and how interventions were evaluated should be in the main body of the paper as this was their stated aim. Some of this was in the discussion and should be moved to the results section.

Other Revisions

Background

• Are there more updated statistics for natural-disasters that can be referenced – current statistics are from 15+ years ago. For example, Food and Ag of UN notes that in 2023 there were still about 400 natural disaster events per year: https://openknowledge.fao.org/server/api/core/bitstreams/069ceb86-59b2-4b6e-90e0-b7bd26a58c76/content

Similarly the 2023 UN Disaster report found 326 major disasters, however substantial increases in deaths and economic impacts: file:///C:/Users/nedgar/Downloads/2023-global-natural-disaster-assessment-report.pdf

• Page 5 Line 14, I think there may be an incomplete sentence–“ Disasters, whether nature-related or human-induced, overwhelm the local capacities more often than not and render

communities seek external assistance in the form of human, financial, or material resources". The bolded portion is not complete.

• Page 5 Line 34 typo: "are the major two organizations that depend…"

• Page 6 line 5 – describes 'humanitarian crisis' but earlier referred to as "nature or human made disaster". I would maybe start with what's included in a humanitarian crisis.

Methods

• Page 8 line 17 – the PRISMA-SCr checklist was included, this should be referred to here as an additional document.

• Page 9 Line 39 - As the search was conducted more than 1 year ago, I would recommend updating the search

• Page 9 Line 39-46 - Was the search strategy peer reviewed (for example following PRESS guidance)?

• Page 9 Line 39-46 (and supplement) - It is not clear to me why the same search strategy was not executed across databases. A short justification for this approach would improve this section (for example were changes only made to accommodate the database function).

• Page 10 Line 7 – If the authors could clarify here which stages included 2 reviewers (for example "two researchers independently completed title-abstract and full-text screening"). The way it is currently worded makes it unclear whether it was both stages or only the full-text.

• Page 11 – I am unclear why it was not possible to engage people with lived experience in any aspect of the study. Given the importance of engaging people with lived experience, I would appreciate some elaboration here.

Results

• Page 11 line 39-40: The authors note that 11 papers were volunteers only and the remainder included mixed samples of volunteers and other professionals. Was there a threshold for inclusion (for example did papers have to include at least 50% of volunteers to be considered as focusing on volunteers)? The exclusion criteria try to exclude papers that focused on other populations that may face PTE through their occupation, however this makes it appear that those may have been included. If a threshold was not used, can the authors please provide a justification for their inclusion and how these would be interventions targeting volunteers.

• Page 14 line 46-48: The authors state "all interventions were found to be more or less effective in improving the mental health conditions". This statement is very vague. I would suggest either: (a) removing this statement as assessing effectiveness was not a stated aim; or (b) clarifying this statement to provide an adequate level of detail. It is unclear which interventions were effective, were not effective, whether any results were significant.

Discussion

• Page 15-16 -Discussion on recent growth of evidence and Factors to be considered - I would recommend a re-write of these sections. These sections do not appear to summarize any of the study findings in relation to the aim of the study (which is about PTSD interventions). These sections seem to focus on risk factors for developing PTSD, exposure to PTEs and information about the organizations themselves. I would recommend re-aligning these sections with the objectives of the study focusing on the interventions identified.

• Page 17 Line 34-39: I am unclear why cost effectiveness is being brought into the discussion at this stage. No mention was made of assessing cost effectiveness as an aim of this review, so I am not sure it is relevant here.

• Page 17 Line 43-51 – This summary is a narrative synthesis that belongs in the results, it is not a discussion.

• Page 18 Lines 36-18– Evaluation of the intervention: This should be in the results. The challenges with these outcome measures etc. can then be discussed here and how it impacts our interpretation of the intervention effectiveness, but the initial evaluation should be described in the results.

• Page 19 Line 47-Page 20 Line 12 – This appears more as background information. I would condense this and link into the following paragraph which offers a clearer relationship to the aims of the study.

Figures

• Figure 1b and c do not really add anything to the manuscript as these are already clearly described elsewhere. I would include figure 1a as it provides a clear picture of the types of evidence identified.

VERSION 1 - AUTHOR RESPONSE

Reviewer: 1

1. The **consistency** with some psychotraumatic terminologies may benefit from a revision. For example, P3 L18 “developing stress”, P7 L20 “acute stress”, P8 L9 “Early interventions to address mental well-being or acute stress”, P14 L14 “could trigger stress”. It is advised to be consistent with the concepts of acute stress reaction (ICD), disorder (DSM), or using the term stress response (when describing the normal physiological phenomenon).

- *To fix the consistency of terminologies throughout the manuscript, the terms “acute stress” and “stress response” have been used to refer to the disorder and the normal physiological phenomenon, respectively. Thank you for pointing out the concern.*

2. To improve the readability, it is advised to add an **additional column in Table 1** P13, of either the **title** or the study hypothesis for each of the included articles instead of keeping this information available only in the supplement materials.

- *A “Title” column has been added to Table 1. Thank you for the suggestion.*

3. References number **53, 54, and 55 don’t focus on PTSD** per se yet they are included. This is a call for authors to either revise their inclusion/exclusion criteria or limit those with sole focus on PTSD to peer-reviewed articles. Later in their review, the author states similar conclusions “The outcome of interest was not always PTSD. In some cases, these interventions aimed to alleviate stress, depression, and anxiety, as well as strengthen resilience, coping, and psychosocial well-being, which would in turn help to prevent PTSD”.

- *Thank you for pointing out this major discrepancy. The aim and the eligibility criteria of the study have been revised to include all kinds of mental health interventions for humanitarian volunteers. The search strategy has also been amended. We repeated the whole screening and selection process from scratch to include two new documents in the final inclusion list. The manuscript has been revised accordingly.*

4. The authors are advised to **revise their extractions to match the exact conclusions** of the included articles. For example, Thormar et al., in Table P 38 L 31 (is not actually reference number 30 but number 24!), doesn’t include group sessions for preparation. Furthermore, it concludes that there is no association between PTSD and training, but only with safety, referencing the regression table in that article. Thormar et al.’s study hasn’t investigated the difference between those who were trained and those who were not. In conclusion, that study has no sufficient assumptions to make any interference of training as an intervention. “Although we expected the more experienced volunteers to have lower symptoms, this was not borne out in the regression analysis. The same applied to those who felt less trained. However, preparation in terms of good information about the situation, quality of equipment to be used, and proper safety measures in place were important,” citing that article itself.

- *We agree that some of the extractions in the last column of the supplemental table did not match the documents because of poor summarizing or rephrasing of the concluding remarks. We apologize for that and have revised the above-mentioned column, trying not to alter the interpretations of the findings this time.*

5. The section “**Factors to be considered P16 L40” doesn’t clearly serve the purpose of the review**. I encourage the authors to revise the links between these factors and the interventions for PTSD for volunteers if they decide to keep it. The personality trait screening proposal looks discriminatory and out of context in humanitarian settings. Authors are requested to bring evidence to support it if they recommend that for humanitarian organisations. Based on the above comment, it might make more sense to discuss this section after the interventions themselves, making some recommendations for practice.

- *We agree that we went out of the aim and scope of this review when we discussed things like associated factors, effectiveness of interventions, and their financial aspects. Moreover, revising the aim of the study made certain parts of the discussion section redundant. We have decided to either remove or realign them, restructuring the discussion section. We thank you for pointing out this concern.*

6. In P18 L30, the article argues that “treating PTSD is quite tedious and lengthy, often not ending with satisfactory results unless intervention is made at an early stage”. The authors

don't bring up-to-date evidence in support of this statement. The diagnosis of PTSD cannot be made before 1 month of exposure (ICD-11), and there are quite decent discrepancies in the effectiveness of early interventions in the literature. The reference provided is from 25 years ago, and the other one only looked at early interventions to prevent, not at the treatment of PTSD!

- *We have removed that part of the discussion section for reasons mentioned above in point 5.*

7. In the nature of interventions in P18, the authors argue that most organisations would prefer post-exposure interventions due to a lack of time and resources in preparing volunteers. I am unaware of any even anecdotal discussion favouring post-exposure intervention over the preparedness of volunteers for resource reasons as these interventions are also costly and require considerable levels of coordination and follow-up. Most probably this interference is attributable to publication bias, as it is difficult to evaluate the preparedness programmes, Thormar et al.'s article is a good example of such. The authors are encouraged to bring evidence supporting their argument.

- *We agree that using the term "prefer" had no basis and it could be a wrong assumption. We have revised that part as we could not find any evidence showing any inclination of the organizations towards post-exposure interventions. Thanks for raising the concern.*

8. In their argument around the nature of interventions in P18, the authors may also wish to **consider bringing ethical and duty-of-care perspectives** to complement their argument, not only to point out the cost-effectiveness part.

- *We have removed that part of the discussion section for reasons mentioned above in point 5.*

9. Contrary to the inclusion criteria of this review, the PFA approach discussed in P18 and 18 doesn't aim to prevent PTSD but rather aims to address the **common mental health conditions and wellbeing after exposure to PTEs**.

- *The PFA approach should now fit the study after revising the aim and the eligibility criteria.*

10. In evaluating the interventions, the article misses the opportunity to **discuss the components of these interventions** rather than listing them, and what makes them special for volunteers. There is little discriminatory explanation of, for example, if PFA or CISM are different for volunteers over paid staff or not, what components of preparation for the work are recommended, who provides the interventions and at what time from the exposure and does each of these interventions include and how do they differ from each other… etc. The authors are encouraged to bring these aspects into their discussion.

- *We agree that discussing the individual components of the reviewed interventions could be quite interesting and insightful. We had tried it initially, but the interventions in question are so heterogeneous that it proved very difficult to discuss and compare them individually. Moreover, as this study focused on how they were evaluated rather than how effective they were, we skipped discussing the components of the interventions and focused more on the general characteristics. Perhaps reviewing a*

specific kind of interventions would be a better scope for an in-depth analysis of their components.

11. In the same section “evaluating the interventions”, the part on DSM, rather than ICD criteria looks out of the scope of the study as this review doesn’t compare the discrepancy of diagnostic tools for PTSD. Most of the included studies compare the symptoms, in terms of scores pre- and post-intervention rather than prevalences, in terms of cut-offs. Unless the authors bring examples of the diagnostic in PTSD (in terms of prevalence % before and after intervention), this argument could be made redundant.

- *This part of the discussion section has been removed after shifting the focus of the study from PTSD to mental health.*

12. In P 19 L2, the authors bring an argument about the perception of volunteers around the intervention as a potential contributor to the actual effectiveness. Even when 2 of the included articles had addressed this matter, **there is no explanation of how so.** Authors are encouraged to elaborate on this matter in light of included articles.

- *We had already looked for evidence in support of this matter during preparing the manuscript. We could only find that individual perceptions contributed to the development of PTSD, from which we only assumed that it could play a role in the effectiveness of the interventions as well. This is a potential link to explore. We have now added it as a suggestion.*

13. The paper would benefit from a summary table of these interventions **before, peri exposure and after exposure interventions**.

- *We have arranged the rows of the “Summary of the reviewed interventions” table according to the timing of the interventions. We hope that it will suffice. Thank you for the comment.*

14. After reading the whole manuscript, it seems like it looks at interventions to address the **adverse mental health consequences of PTEs** rather than PTSD among humanitarian volunteers. The outcomes of the included articles, particularly with prevention, go beyond PTSD per se. The authors are encouraged to refine their aim statement accordingly.

- *The aim and the eligibility criteria of the scoping review have been modified to include all kinds of mental health interventions, and the manuscript has been revised accordingly.*

Reviewer: 2

1. The manuscript does not seem to focus on humanitarian volunteers or specifically PTSD interventions.

a. The Summary of Interventions supplementary table indicates that 7/12 of the interventions identified were not available to volunteers. How did these papers meet eligibility criteria to be included? I am not sure that these can be considered “interventions for humanitarian volunteers” if half the interventions were not available to volunteers. The authors should clarify or revise.

b. In the results, the authors state that the outcome of interest "was not always PTSD", however their inclusion criteria state that "documents that explored any other health condition beyond or unrelated to PTSD would be excluded". This should be clarified further. The authors state that these other outcomes "would in turn help to prevent PTSD", however I am not sure what evidence is provided to support this. As the author's stated aim is to describe PTSD interventions, the results should either: be focused on PTSD specifically or more clearly draw the link between the non-PTSD outcomes and how this impacts PTSD.

- *It is true that the initial focus of the research was PTSD only. However, we gradually included other mental health-related issues as well after retrieving interesting and informative documents from the search and tried to link them to PTSD. We apologize that we did not modify the aim and the eligibility criteria of the study accordingly. We have now revised the manuscript to focus on all kinds of mental health conditions. We revised the search strategy and repeated the screening and selection process from scratch to include two new documents in the final inclusion.*
  A few of the interventions were only being tested, while some were suggested in the form of guidelines. When we said they were "not available" for volunteers, we meant that they were not practically implemented in the field yet. But they still had the potential to help the volunteers, which is why we decided to include them as well. We have rephrased relevant sentences in the manuscript to clarify the matter. We truly thank you for raising these concerns.

2. The results do not highlight what types of interventions were identified or provide any sort of narrative that addresses their research question. The authors note that this is in the "supplemental material" but a high-level overview of the interventions identified and how interventions were evaluated should be in the main body of the paper as this was their stated aim. Some of this was in the discussion and should be moved to the results section.

- *We agree that the most important part of the data was presented in the "Summary of the reviewed interventions" table as a supplemental file. We chose not to narrate them in sentences in the results section, as presenting them in a table was suggested to be a more comprehensible way for the reader. Then, we had initially placed it in the main body of the manuscript. But we were advised to put it in a supplemental file as it was too big for the main article according to the journal's policy. We hope you would kindly consider our explanation.*

Other Revisions
Background
• Are there more updated statistics for natural-disasters that can be referenced – current statistics are from 15+ years ago. For example, Food and Ag of UN notes that in 2023 there were still about 400 natural disaster events per year:
https://openknowledge.fao.org/server/api/core/bitstreams/069ceb86-59b2-4b6e-90e0-b7bd26a58c76/content
Similarly the 2023 UN Disaster report found 326 major disasters, however substantial increases in deaths and economic impacts: file:///C:/Users/nedgar/Downloads/2023-global-natural-disaster-assessment-report.pdf

- *Updated statistics from more recent references have been added. We thank you for sharing the links.*

• Page 5 Line 14, I think there may be an incomplete sentence–"Disasters, whether nature-related or human-induced, overwhelm the local capacities more often than not and render communities seek external assistance in the form of human, financial, or material resources". The bolded portion is not complete.

- *We have fixed the grammar and the typos where necessary. We apologize for overlooking these mistakes.*

• Page 5 Line 34 typo: "are the major two organizations that depend…"

- *We have fixed the grammar and the typos where necessary. We apologize for overlooking these mistakes.*

• Page 6 line 5 – describes 'humanitarian crisis' but earlier referred to as "nature or human made disaster". I would maybe start with what's included in a humanitarian crisis.

- *We replaced the word "crisis" with "disaster" where applicable to keep the terms consistent throughout the manuscript. Thank you for raising the concern.*

Methods

• Page 8 line 17 – the PRISMA-ScR checklist was included, this should be referred to here as an additional document.

- *The PRISMA-ScR checklist has been provided as a supplemental file and mentioned accordingly in the methods section. Thank you for the suggestion.*

• Page 9 Line 39 - As the search was conducted more than 1 year ago, I would recommend updating the search

- *The search has been updated as mentioned above. We thank you for this important suggestion.*

• Page 9 Line 39-46 - Was the search strategy peer reviewed (for example following PRESS guidance)?

- *This research was done as a thesis at a university. The search strategy has been peer-reviewed in the sense that it was presented and discussed in front of a class where multiple experts in this field were also present. Then, it was revised based on the discussions and their suggestions. Thank you for raising the concern.*

• Page 9 Line 39-46 (and supplement) - It is not clear to me why the same search strategy was not executed across databases. A short justification for this approach would improve this section (for example were changes only made to accommodate the database function).

- *As you have correctly stated, the search queries had to be tailored to accommodate the different search systems of different databases. In cases where the search returned a huge number of results, we had to use filters to narrow the search down. Moreover, searching in databases is not a precise task. We hoped that using four different bibliographic websites would give us a complete and relevant list of references even if the search queries were slightly different for them. It is difficult to explain it in short, but a justification has now been added to the methods section.*

• Page 10 Line 7 – If the authors could clarify here which stages included 2 reviewers (for example “two researchers independently completed title-abstract and full-text screening”). The way it is currently worded makes it unclear whether it was both stages or only the full text.

- *All the stages were completed by two individual researchers, and then the results were cross-matched. We have added a couple of lines to the methods section to make the process clearer.*

• Page 11 – I am unclear why it was not possible to engage people with lived experience in any aspect of the study. Given the importance of engaging people with lived experience, I would appreciate some elaboration here.

- *As mentioned above, this study was a thesis project at a university. Due to the time and resource constraints of a thesis student, a secondary research methodology was chosen. But we fully agree that primary qualitative research recording the lived experiences of human participants would be valuable, and so we have added it as a recommendation in the relevant section of the manuscript. Thank you for raising this concern.*

Results

• Page 11 line 39-40: The authors note that 11 papers were volunteers only and the remainder included mixed samples of volunteers and other professionals. Was there a threshold for inclusion (for example did papers have to include at least 50% of volunteers to be considered as focusing on volunteers)? The exclusion criteria try to exclude papers that focused on other populations that may face PTE through their occupation, however this makes it appear that those may have been included. If a threshold was not used, can the authors please provide a justification for their inclusion and how these would be interventions targeting volunteers.

- *There was no threshold for the number of volunteers during the inclusion of documents. As a scoping review, our aim was to capture the wider picture by including all kinds of interventions, no matter how small the number of volunteers was. Our justification was that an intervention applied to a small number of volunteers (with or without other professionals involved) might also have the potential to be used for a bigger number of volunteers. Of course, it would require further research. We excluded papers only if they solely focused on other populations except volunteers. We have now revised the exclusion criteria to make it simpler and clearer. Thank you for raising this question.*

• Page 14 line 46-48: The authors state “all interventions were found to be more or less effective in improving the mental health conditions”. This statement is very vague. I would suggest either: (a) removing this statement as assessing effectiveness was not a stated aim; or (b) clarifying this statement to provide an adequate level of detail. It is unclear which interventions were effective, were not effective, whether any results were significant.

- *We agree that the statement exceeds the scope of this review. The same could be said for a couple of other statements as well. We have perused the manuscript to remove or edit such statements. Thank you for pointing it out.*

Discussion

• Page 15-16 -Discussion on recent growth of evidence and Factors to be considered - I would recommend a re-write of these sections. These sections do not appear to summarize any of the study findings in relation to the aim of the study (which is about PTSD interventions). These sections seem to focus on risk factors for developing PTSD, exposure to PTEs and information about the organizations themselves. I would recommend re-aligning these sections with the objectives of the study focusing on the interventions identified.

- *We agree that we went out of the aim and scope of this review when we discussed things like associated factors, effectiveness of interventions, and their financial aspects. Moreover, revising the aim of the study made certain parts of the discussion section redundant. We have decided to either remove or realign them, restructuring the discussion section. We thank you for pointing out this concern.*

• Page 17 Line 34-39: I am unclear why cost effectiveness is being brought into the discussion at this stage. No mention was made of assessing cost effectiveness as an aim of this review, so I am not sure it is relevant here.

- *We have removed this part also due to the reasons mentioned above in the previous response.*

• Page 17 Line 43-51 – This summary is a narrative synthesis that belongs in the results, it is not a discussion.

- *The mentioned part of the discussion has been moved to the results section. Thank you for the suggestion.*

• Page 18 Lines 36-18– Evaluation of the intervention: This should be in the results. The challenges with these outcome measures etc. can then be discussed here and how it impacts our interpretation of the intervention effectiveness, but the initial evaluation should be described in the results.

- *The mentioned part of the discussion has been moved to the results section. Thank you for the suggestion.*

• Page 19 Line 47-Page 20 Line 12 – This appears more as background information. I would condense this and link into the following paragraph which offers a clearer relationship to the aims of the study.

- *The mentioned part of the discussion has been realigned. We appreciate the suggestion.*

Figures

• Figure 1b and c do not really add anything to the manuscript as these are already clearly described elsewhere. I would include figure 1a as it provides a clear picture of the types of evidence identified.

- *As Figure 1 and Table 1 present the same data, we are keeping the table only. Thank you for the comment.*

VERSION 2 - REVIEW

**Reviewer**	**2**
**Name**	**Edgar, Nicole**
**Affiliation**	**Ottawa Hospital Research Institute, Clinical Epidemiology Program**
**Date**	**02-Jun-2025**
**COI**	

Thank you to the authors for their time and efforts to respond so thoroughly to the initial feedback. The narrative summary of results is much improved and provides a clear overview of what was found in the review. Thank you to the authors for this change.

I do still have some concerns that their original protocol was not followed, per the authors' response to comments. However, with some additional revisions, I feel this concern will be addressed.

1. I think the authors should make clear that the aim of the paper changed during the conduct of the project and was expanded from PTSD to mental health more broadly. Setting an a priori objective is an important part of the review process and so, for transparency, I think it would be appropriate to describe this change in the methods section.

2. More information is needed on how the search strategy was updated and implemented:

a. It is not described in the paper that the search strategy was updated or on what date the updated search was run. This should be clarified.

b. It is not clear whether this updated search was run in addition to or in place of the old search strategy. The search strategy provided in the initial review is completely different from the R1 version provided now. This should be clear if two separate search strategies were used, and if so, both should be included.

c. Was the search with the initial terms updated from January 2024 or was the updated search conducted only using the new search terms? If papers were included using the first version of the terms, this search should be re-run and specified in the paper as well.

One minor stylistic choice (which the authors can use or not) – Page 11, for types of studies I would suggest re-ordering to be highest number of studies to lowest (so 4 RCTs, 3 cross-sectional etc.).

VERSION 2 - AUTHOR RESPONSE

Reviewer 2:

Miss Nicole Edgar, Ottawa Hospital Research Institute

Comments to the Author:

Thank you to the authors for their time and efforts to respond so thoroughly to the initial feedback. The narrative summary of results is much improved and provides a clear overview of what was found in the review. Thank you to the authors for this change.

I do still have some concerns that their original protocol was not followed, per the authors' response to comments. However, with some additional revisions, I feel this concern will be addressed.

1. I think the authors should make clear that the aim of the paper changed during the conduct of the project and was expanded from PTSD to mental health more broadly. Setting an a priori objective is an important part of the review process and so, for transparency, I think it would be appropriate to describe this change in the methods section.
    - *Thank you for the detailed comments. We have tried to mention all the required information in the relevant sections in a concise way. We hope the changes will add to the transparency of the methodology.*
2. More information is needed on how the search strategy was updated and implemented:
    a. It is not described in the paper that the search strategy was updated or on what date the updated search was run. This should be clarified.
        - *The dates of the updated search have been mentioned in the relevant section.*
    b. It is not clear whether this updated search was run in addition to or in place of the old search strategy. The search strategy provided in the initial review is completely different from the R1 version provided now. This should be clear if two separate search strategies were used, and if so, both should be included.
        - *We developed a new search strategy from scratch, keeping the old search results also. Then we compared the old and the new results, which helped us to reduce the risk of missing important documents.*
    c. Was the search with the initial terms updated from January 2024 or was the updated search conducted only using the new search terms? If papers were included using the first version of the terms, this search should be re-run and specified in the paper as well.
        - *As we developed the new search strategy from scratch, everything was up to date. Replacing the terms related to PTSD with mental health-related terms was the only major change. The rest of the terms were more or less the same, with minor changes that should not affect the search results. The searching process in academic databases is not as precise as we would like it to be.*

Hence, we kept both old and new results to reduce the risk of missing important and relevant documents. We should also mention that we tried different search strategies in both cases and kept the ones with higher results.

3. One minor stylistic choice (which the authors can use or not) – Page 11, for types of studies I would suggest re-ordering to be highest number of studies to lowest (so 4 RCTs, 3 cross-sectional etc.).
   - *Thank you for the suggestion. It makes sense and we have gladly accepted it.*
